# Ingestion of a Cold Temperature/Menthol Beverage Increases Outdoor Exercise Performance in a Hot, Humid Environment

**DOI:** 10.1371/journal.pone.0123815

**Published:** 2015-04-09

**Authors:** Than Tran Trong, Florence Riera, Kévin Rinaldi, Walid Briki, Olivier Hue

**Affiliations:** Laboratoire ACTES—EA 3596, Université des Antilles et de la Guyane, Campus de Fouillole, Point à Pitre, France; St. Joseph's Hospital and Medical Center, UNITED STATES

## Abstract

**Purpose:**

A recent laboratory study demonstrated that the ingestion of a cold/menthol beverage improved exercise performance in a hot and humid environment during 20 km of all-out cycling. Therefore, the aim of this study was to determine whether the ingestion of cold water/ice-slurry with menthol would improve performance in hot and humid outdoor conditions.

**Methods:**

Ten trained males completed three trials of five blocks consisting of 4-km cycling and 1.5-km running. During warm-up, every block and recovery, the athletes drank 190 ml of aromatized (*i*.*e*., with 0.05 mL of menthol) beverage at three temperatures: Neutral (ambient temperature) (28.7°C±0. 5°C), Cold (3.1°C±0.6°C) or Ice-slurry (0.17°C±0.07°C). Trial time, core temperature (T_co_), heart rate (HR), rate of perceived exertion (RPE), thermal sensation (TS) and thermal comfort (TC) were assessed.

**Results:**

Ice-slurry/menthol increased performance by 6.2% and 3.3% compared with neutral water/menthol and cold water/menthol, respectively. No between-trial differences were noted for T_co_, HR, RPE, TC and TS was lower with ice-slurry/menthol and cold water/menthol compared with neutral water/menthol.

**Conclusion:**

A low drink temperature combined with menthol lessens the performance decline in hot/humid outdoor conditions (*i*.*e*., compared with cold water alone). Performances were better with no difference in psycho-physiological stress (T_co_, HR and RPE) between trials. The changes in perceptual parameters caused by absorbing a cold/menthol beverage reflect the psychological impact. The mechanism leading to these results seems to involve brain integration of signals from physiological and psychological sources.

## Introduction

Aerobic performance is decreased in tropical climates because the thermal stress exceeds the evapotranspiration process [15]. Although the processes involved in the alteration of performance are unclear, several mechanisms have been proposed, including thermoregulatory anticipation [14], decreased power output [22] and cardiovascular adjustments [20]. Whatever the mechanisms may be, the clear consensus is that the tropical climate is deleterious for those who are unacclimated [34], acclimated [34], and even native to the climate [33].

Recently, drinking cold water/ice-slurry [16, 18], swilling an L (-) menthol solution [21], and the ingestion of combined cold water/ice-slurry and menthol [24] were reported to be beneficial strategies for endurance performance in the heat. The study of Mündel et al. [21] showed a decrease in exercise-induced exhaustion when athletes periodically swilled an L (-) menthol solution of 0.01%. Because L (-) menthol creates a cooling sensation in the mouth [11], it was suggested that the thermal stimulation signal in the mouth when drinking cold mint is transmitted to the reward-motivating regions of the brain, thereby extending the exercise duration [21]. Further, it was shown that the cooling sensation in the intra-mouth was strengthened with each subsequent inhalation of cool ambient air or by cold drinking after gargling with an L (-) menthol solution [11]. Very recently, Riera et al. [24] indicated that L (-) menthol combined with cold water/ice-slurry improved exercise performance in hot and humid environments during 20 km of all-out cycling without changes in HR or T_co_. These authors suggested that menthol might impair the heat perception process by affecting the activity of hypothalamic thermoreceptors [24].

Although studies on exercise performed in valid outdoor conditions are particularly important to understanding how physiological responses are affected [9], most of the studies cited above were performed in a laboratory and several environmental factors could not be taken into account. For example, in natural sunlight, the human body receives the radiant heat load directly, which can increase skin temperature [4,13] and thermal sensations [13] and reduce the temperature gradient between the skin and the core; this in turn accelerates the rate of heat storage and reduces the output power [31]. Furthermore, thermal regulation is also affected by the speed of air flow around the body (which is influenced by the wind velocity) [26, 30], which can positively affect endurance performance through a decrease in thermal stress [26], and this air flow may also positively affect the cold stimulus in the mouth while breathing.

Therefore, the aim of this study was to determine whether the positive results (an increase in exercise performance) for cold water/ice-slurry with L (-) menthol ingestion recently found in the laboratory could be extended to other sports (cycling and running) or exercise modalities in outdoor conditions.

## Methods

### Subjects

Ten heat-acclimated; trained (*i*.*e*., living and training in Guadeloupe) male cyclists and triathletes (age: 41±17 years, height: 179±9 cm, body mass: 73±7 kg, maximum aerobic capacity (VO_2max_): 59±11mL.min^-1^.kg^-1^,peak power output at max: 335±48 W) participated in the study. The athletes were training at least 10 hours per week at the time of study. The study was approved by the Ethics Committee of the Medicine and Sport Center in Guadeloupe (Ministry of Youth and Sports) and the Ethics Committee of the Training and Research in Sport Science Unit in Guadeloupe (Ministry of Higher Education and Research). All athletes completed a medical screening questionnaire and gave written informed consent prior to the study, which was approved by the University Ethics Committee and conducted according to the Declaration of Helsinki.

### Preliminary measurements

On the athletes’ first visit to the laboratory, VO_2max_was measured during an incremental exercise test on an electronically braked cycle ergometer (TECHMED, TM 4170, Besançon, France). The initial workload was 30 W and increased by 30 W every minute until volitional fatigue. Gas exchange was measured throughout the entire test (ZAN Ferraris, Cardiorespiratory System, Oberthulba, Germany). VO_2max_was reached when two of the following criteria were met: (1) VO_2_ did not increase with an increase in intensity, (2) heart rate (HR) was within 10 beats.min^-1^ of the age-predicted maximum of 220—age, and (3) the respiratory exchange ratio (RER) was greater than 1.05.

### Experimental design

The experimental trials were separated by 7 days and were undertaken in a randomized crossover design. The athletes were also asked to limit exercise to 60 minutes of light-intensity exercise the day before each trial. At the start of the trial days, the athletes consumed a standard breakfast that included food and 600 mL of beverage. The trials began at the same time of day for each athlete (between 12:00 and 15:30) to control for circadian variations in core temperature (T_co_) and digestion. During the trials, the athletes wore cycling shorts, a cycling jersey, socks and shoes.

### Experimental procedures

The experimental trials were performed in an outdoor cycling stadium in the hot/humid conditions of Guadeloupe, French West Indies (WBGT: mean±SD outdoor temperature: 27.6°C±0.8°C, dry bulb temperature: 32.5±1.2°C; 57%±0.05% RH and wind: 25.9 km.h^-1^±0.7 km.h^-1^). HR was monitored continuously using a portable telemetry unit (Suunto Memory Belt, Suunto, Vantaa, Finland) with recording every 10 seconds, and the data were analyzed with Suunto software. T_co_ was assessed via the gastrointestinal temperature using ingestible temperature measurement pills (CorTemp, HQ, Inc., Palmetto, FL, USA). The athletes were instructed to ingest these pills 6 to 8 h before all experimental trials to ensure the pill was out of the stomach, thereby avoiding variability in T_co_ due to pill movement or fluid/food consumption.

The experimental trial included 15 min of warm-up with cycling at a freely chosen cadence, followed by five blocks of 4-km cycling and 1.5-km running for each block with the fastest possible time, and then 15min of recovery at a free cadence. There were two transition periods when the participants replaced their cycling shoes with running shoes or the reverse: (1) 60 seconds at the end of 4-km cycling to the start of 1.5-km running in each block and (2) 90 seconds at the end of 1.5-km running of the previous block to the start of 4-km cycling in the next block (between blocks).

During the experimental trials, the athletes were asked to drink 190 mL of a randomly assigned beverage during the 15-min warm-up, during the running segment in each block, and during the 15-min recovery. The three experimental trials differed as follows: a menthol aroma beverage was ingested at one of three temperatures: (1) Neutral (28.7°C±0.5°C), (2) Cold (3.1°C±0.6°C) or (3) Ice-slurry (0.17°C±0.07°C). The L (-) menthol beverages used a 0.025% natural menthol aroma (% vol: 86.0% ± 1.0; dosage recommendation by manufactory: 5/g/L) (Robertet, Grasse, France). The ice-slurry was produced with an ice-slurrymachine (Brema, GB 902A, Professional Slush Machine, Ice Makers, Germany). Although ice expands in volume, we carefully ensured that the volume of ice-slurry (in mL of water) was precisely the same as the volume of cold water. The temperature of each beverage was measured with a digital thermometer (YSI 409B, Yellow Springs Instruments, OH, USA). A straw with a 1.5cm diameter was connected to the lid of the water bottle to aid ingestion of the ice-slurry.

### Measurements

Perceived exertion (RPE), perceived thermal sensation (TS), and perceived thermal comfort (TC) were recorded, as thermal perception and physiological strain during exercise are known to affect pacing strategy and performance [8]. During the experimental trials, T_co_ was measured before and after warm-up, at the end of every 4km of cycling and every 1.5km of running, and after the recovery phase. Before and after warm-up and at the end of each block (at the end of 1.5-km running) of the trial, the athletes were asked to rate their perceived TS and perceived TC, respectively, on a 7-point scale (ranging from “extremely cold” (1) to “extremely hot” (7)) and a 4-point scale (ranging from “comfortable” (1) to “very uncomfortable” (4)) adapted from Hodder and Parsons [13]. They were asked to rate their perceived exertion according to the 15 grades of Borg’s perceived exertion scale [2] at the end of each block. Nude body mass was assessed (± 0.1 kg) before and after each session with an electronic scale (Terraillon Pop, France). Hydration status throughout the experimental trials was estimated by changes in nude body mass. During the session, the athletes drank a total of 1330 mL of beverage.

### Statistical analyses

We tested for normality using Skewness and Kurtois tests, with acceptable Z values not exceeding +1 or -1. Once the assumption of normality was confirmed, parametric tests (Mauchly'ssphericity test) were performed. To examine our hypotheses regarding the changes in T_co_, performance, HR, TS, TC and RPE as a function of time, a 3 × 5 repeated measures ANOVA (Drink Temperature [Neutral *vs*. Cold *vs*. Ice-slurry] × Time Period [T1, T2, T3, T4, and T5]) was conducted, and Tukey’s post hoc test assessed the differences between adjacent time periods, with significant differences reflecting abrupt changes in performance, and between distant periods, with significant differences reflecting only gradual changes [3]. Data analysis was performed using the Statistical Package for Social Sciences (SPSS version 19.0). Significance was set at the P< 0.05. All data are presented as mean ± SD.

## Results

### Performance

The global performance was affected by Drink Temperature (P<0.03), with the performance with Ice-slurry (4289±190s) significantly better than with Cold (4436±171s, P<0.002) or Neutral)(4572±423s, P<0.04). The global performance was also affected by Time Period (P<0.02) and the Time Period x Drink Temperature interaction (P<0.007) (S1 Fig).

When expressed by block (cycling + running), the performance was significantly affected by Drink Temperature (P<0.004), with Ice-slurry (858±7s) greater than Cold (887±32s, P<0.007) and Neutral (914±92s, P<0.008). The block performance was also affected by Time Period (P<0.007) and the Time Period x Drink Temperature interaction (P<0.004) (S2 Fig).

Although there was no exercise (i.e., cycling or running) x condition interaction (P>0.05), both types of exercise being affected by the Time Period x Drink Temperature interaction (*i*.*e*., P<0.004 and P<0.03 for cycling and running, respectively), the mean time with Ice-slurry was better than Neutral in cycling (429±5s vs. 455±22 s for Ice-slurry and Neutral, respectively, P<0.05) and better than Cold in running (*i*.*e*., 430±7s vs. 450±18s for Ice-slurry and Cold, respectively, P<0.006).

### Core temperature (T_co_), heart rate (HR)

There was no significant difference in the mean T_co_ noted in the trials before exercise (for Neutral: 36.9°C ± 0.5°C, Cold: 37.3°C ± 0.5°C, Ice-slurry: 37.3°C ±0.3°C, P>0.05). During the trials, T_co_ increased over time (P<0.0001) with no significant difference between trials (P>0.05) (S3 Fig). There were no significant differences in T_co_ for cycling, running or blocks (cycling + running) between trials (P>0.05) (S1 Table). However, T_co_ was significantly affected by Time Period in the trials (P<0.0001).

HR increased significantly during the trials from warm-up to the end of exercise (P<0.0001), but there was no significant difference in the mean HR between trials (P>0.05) (S4 Fig). There were no significant differences in HR for cycling, running or blocks (cycling + running) between trials (P>0.05) (S1 Table). However, HR was significantly affected by Time Period in the trials (P<0.0001).

### RPE, thermal sensation, thermal comfort

TS was significantly lower in the Ice-slurry (3.5±0.8) and Cold (3.4±1.0) conditions than in Neutral condition (4.0±1.2) (P<0.05 and P <0.02, respectively)(S5A Fig). There were no significant differences in TC (for Neutral: 2.3±0.9, Cold: 2.1±0.8, Ice-slurry: 2.0±0.8, P>0.05) (S5B Fig) or RPE (P>0.05) (S5C Fig), with all of them increasing continuously with Time Period (P<0.0001).

### Environmental conditions, weight and hydration status

There was no difference in the mean temperature (WBGT: 27.6°C±0.8°C, dry bulb temperature: 32.5°C±1.2°C, P>0.05), relative humidity (57%±0.05%, P>0.05) or wind speed (25.9 km.h^-1^±0.7 km.h^-1^, P>0.05) in the area where the three trials took place and the athletes’ body weight loss did not differ between trials (for Neutral: 2.3±0.9kg, Cold: 2.2±1.3kg, and Ice-slurry: 2.2±0.7kg, P>0.05).

## Discussion

The most important results of this study were the following: (1) the ingestion of the ice-slurry/menthol drink increased performance by 6.2% and 3.3% compared with neutral water/menthol and cold water/menthol, respectively. Although not significant, the performance with cold water/menthol ingestion was 3% better than with neutral water/menthol; and (2) despite the improvement in performance, psycho-physiological stress (T_co_, HR and RPE) did not differ between trials.

### Influences of the combination of cold drink/ice-slurry and L (-) menthol on exercise performance

In a laboratory study, Riera et al. [24] showed significantly better performance with the absorption of ice-slurry/menthol and cold water/menthol compared with neutral water/menthol. In the present study, the post-hoc analysis demonstrated significantly better performance with ice-slurry/menthol, but not with cold water/menthol (*i*.*e*., compared with neutral water/menthol). Despite this lack of significance, we believe our results support the findings of Riera et al. [24]. Indeed, a significantly lower performance benefit has been observed with ice-slurry (*i*.*e*., compared with neutral water) in laboratory settings as opposed to field settings. In the laboratory, a significant increase of 6.8% and 2.5% in performance was shown by Ishan et al. [16] and Stevens et al. [29], respectively, but in an actual field setting this increase was only 0.5% in the study of Yeo et al. [35]. Conversely, the decline in performance benefit was not significant in the case of a cold beverage with menthol (i.e., cold water/menthol or ice-slurry/menthol). Riera et al. [24] observed a performance increase of 8.2% and 5.0% with ice-slurry/menthol and cold water/menthol compared with neutral water/menthol, respectively, whereas the rates were 6.2% and 3.0% in our study. We assume that the impact of environmental factors (*i*.*e*., wind and solar radiation) reduced the effects of a cold beverage (i.e., cold water, ice-slurry) on the body but that this impact was attenuated by the cold beverages containing menthol versus no menthol because the cold temperature beverages with menthol elicited both physical and chemical activation of cold receptors. Thus, a low temperature/menthol combination lessens the decline in performance benefit (*i*.*e*., compared with only cold beverage) in outdoor conditions. Moreover, although some studies with ice-slurry absorption in self-paced exercise have failed to show further performance improvement when combined with other strategies, such as external precooling [19, 25] and hyper hydration [25], or an improvement compared with neutral water [23, 28], the 6.2% increase in performance with ice-slurry/menthol demonstrated in the present study was significant.

The best time performance was over-expressed in blocks 4 and 5 (S2 Fig). This is consistent with the results of several studies [16, 24, 29, 35], in which the better performances with ice-slurry or ice-slurry/menthol appeared near the end of exercise.

### Influences of the combination of cold drink/ice-slurry and L (-) menthol

The decrease in T_co_ with the absorption of cold water or ice-slurry, as compared with the absorption of neutral water, is assumed to be the main factor in improved exercise performance [16, 18,27]. However, studies [5, 24] have shown that exercise performance may increase with the absorption of a cold drink without any change in T_co_ and/or HR. It has therefore been suggested that the cooling sensation created in the throat by drinking a cold beverage also contributes to the increase in exercise performance [5, 27]. In our study, T_co_ was not reduced by ingestion of a cold drink with menthol (cold water, ice-slurry), and both the feeling of freshness in the pharynx and the taste of menthol may have contributed to the increase in exercise performance [5,21]. Indeed, some of the same human brain regions involved in detecting temperature were found to also be involved in sensing pleasantness by intra-oral thermal stimulation [12], and the suggestion was made that the pleasant stimulus created by cold absorption may help to maintain central drive and increase motivation for exercise [6]. Moreover, Mündel and [21] showed that the pleasant sensation generated by gargling regularly with a solution of L(-) menthol enhanced exercise performance compared with placebo, but created no difference in the T_co_ between trials.

It has also been hypothesized that a false signal [7] or an incorrect assessment of thermal stress [32] can create a subconscious physiological effect that leads to improved performance. Tyler et al. [32] showed that the application of a cooling collar decreased the neck skin temperature, and exercise performance increased by 6% without an alteration in T_co_, HR, RPE or neuroendocrinological response. The explanation was that the lower neck skin temperature from the cooling collars created signals which overrode inhibitory feedback, thereby giving a false assessment of the body’s thermal status. This was assumed to lead to the selection of a faster pace, and exercise capacity subsequently increased compared with the no cooling condition [32]. We suspect that the effect of cold water and menthol in the oropharyngeal region may have been similar.

In an outdoor environment, the physiological processes (*i*.*e*., thermoregulation) are affected by a number of factors such as wind and solar radiation. During the rest period before exercise (or when athletes move at low speed), the impact of wind on psychological and physiological processes (*i*.*e*., TS, TC, thermoregulation) is not significant [15, 26], whereas solar radiation can cause an increase in skin temperature [4] and thermal sensation [13]. L (-) menthol creates a cooling sensation in the mouth, which is strengthened when combined with a cold beverage [11], creating higher intensities of the stimuli in the throat compared with neutral water/menthol. In addition, Gibson and Noakes [10] introduced the central governor model through which the brain makes calculations in order to determine the optimal pacing strategy and maintain a physiological reserve to enable increased exercise capacity at the end of exercise. According to this model, the afferent input from skin and signals of coolness from drinking cold beverage/menthol (*i*.*e*., cold or ice-slurry) are integrated in the brain and interact. We suspect that sufficiently high intensities of the stimuli in the present study, that is, ice-slurry and menthol, may have overwhelmed the heat stress signals (from skin), whereas the combination of cold water and menthol was insufficient to elicit this effect.

Thus, it is plausible that the ingestion of ice-slurry/menthol created more pleasant sensations and/or an incorrect assessment of thermal stress, based on which the governor region of the brain established an intensity to enable an increase in exercise performance. The central governor model would also explain why the improved exercise performance with ice-slurry/menthol, as opposed to that with cold water/menthol and neutral water/menthol, appeared in the last blocks (4 and 5) (S2 Fig).

### T_co_ and HR

We observed no between-trial difference in T_co_ for the three types of beverage/menthol (*i*.*e*., at 28.7, 3.1 and 0.17°C) during the 15-min warm-up, running in each block, and the recovery (S3 Fig). Previous studies have indicated that the timing of water ingestion is an important factor in determining the reduction in T_co_. The study results of Ihsan et al. [16], Siegel et al. [27] and Lee et al. [18] all indicated that an effective strategy to reduce T_co_ was to ingest a cold beverage during the rest phase before exercise or during the period separating two series of exercise [28]. Conversely, the studies of Riera et al. [24], Burdon et al. [5], Burdon et al. [6], and Lee et al. [17] indicated that the absorption of cool water (i.e., cold water, ice-slurry) during exercise did not reduce T_co_ compared with neutral water. Lee et al. [17] hypothesized a response mechanism to the impact of exogenous heat on the body, whereby cold-water ingestion during exercise cannot reduce T_co._ If such a mechanism exists, we suspect that it is not operational in the rest state before exercise; hence, T_co_ decreases with the ingestion of a cold drink. Instead, we suspect that this putative mechanism is activated only once exercise begins (including warm-up at low intensity), which would explain the lack of difference in T_co_ between trials in our study. Another explanation is that the body produces more heat as performance increases. Thus, the combined effect of increased performance and ice-slurry ingestion led to a constant T_co_ [24].

In this study, the best performance was obtained with ice-slurry/menthol with no difference in HR between trials (S4 Fig). We hypothesize that the ingestion of ice-slurry/menthol provided the brain with false information on heat stress, causing a reflex that decreased blood flow to the skin [7]. This is supported by the study of Riera et al. [24], who suggested that under the influence of menthol, the hypothalamus does not detect increases in T_co_ and thus no inhibitory signal is sent to the motor control centers. If this assumption is correct, blood would not have been redistributed from the core to the periphery, thereby allowing the central blood volume to increase and, thus, improving perfusion to the exercising muscles. Further, the improved central blood would have ensured larger stroke volume; hence, the HR remained constant (the stroke volume not being maintained in the neutral water/menthol and cold water/menthol condition). This would explain the better performance with ingestion of ice-slurry/menthol without any change in HR.

### Sensations

Psychological effects have a powerful influence on performance in a warm environment [8]. In the present study, the ingestion of ice-slurry/menthol and cold water/menthol led to lower thermal sensations compared with neutral water/menthol (S5A Fig). Nevertheless, exercise performance differed only between the ice-slurry/menthol and neutral water/menthol conditions. Teunissen et al. [30] and Barwood et al. [1] hypothesized that RPE, rather than thermal perception, is the primary factor for improving pacing strategy and performance in hot environments, based on Mündel [21] demonstration that periodic gargling with a different concentration of menthol solution compared with placebo resulted in lower rates of perceived exertion. We might have expected lower RPEs for the combination of cold or ice with menthol, which was not the case (S5C Fig). However, our results agree with those of Riera et al. [24], who conducted a laboratory study with similar exercising and beverage conditions. Because RPE is influenced by both environmental, internal factors and exercise intensity, we suspect that the increase in performance (thus in the relative intensity) noted in both the ice-slurry and cold conditions compared with neutral condition may have masked the effect of combined cold/ice-menthol on RPE.

## Conclusions

This study showed that a low drink temperature combined with menthol lessened the performance decline in hot and humid outdoor conditions (*i*.*e*., compared with cold water alone). Performances were better with no difference in psycho-physiological stress (T_co_, HR and RPE) between trials. The changes in perceptual parameters caused by absorbing a cold drink with menthol reflect the impact on psychological factors. The mechanism leading to these results seems to involve brain integration of signals from physiological and psychological sources.

## Perspectives

The results of this investigation showed the benefits of cold beverage/menthol to increase time-trial performance in actual field settings. Furthermore, the ice-slurry equipment is compact [29] and preparation for the athlete is easy. The ingestion of ice-slurry/menthol can therefore be recommended for endurance sports (*i*.*e*., cycling, running) when competitions are held in a hot climate. However, we expect that the benefits of cold beverage/menthol may also be extended further. In fact, previous studies [16, 35] have shown that ice-slurry ingestion before exercise (internal precooling) reduces T_co_, which results in an increase in self-paced performance. The results of Riera et al. [24] and the present work have demonstrated that ice-slurry/menthol ingestion in the course of exercise does not reduce T_co_, although exercise performance increases. Therefore, a strategy of internal precooling through ice-slurry/menthol ingestion to increase self-paced performance in a hot environment should be further examined in future studies.

## Supporting Information

S1 Fig Trial times (seconds) for cycling (C) and running (R) during trials with the ingestion of Neutral water, Cold water and Ice-slurry. Mean values and SD are shown. β,† denote that global performance was affected by Time Period (P<0.02) and the Time Period x Drink Temperature interaction (P<0.007), respectively.
(EPS)

S2 Fig Trial times (seconds) for blocks (cycling+running) during the trials with the ingestion of Neutral water, Cold water and Ice-slurry. ^a^ Significantly different from Neutral water (P<0.05), ^b^ Significantly different from Cold (P<0.05). Mean values and SD are shown. β,† denote that block performance was affected by Time Period (P<0.007) and the Time Period x Drink Temperature interaction (P<0.004), respectively.
(EPS)

S3 Fig Core temperature during warm-up, exercise and recovery for trials with the ingestion of Neutral water, Cold water and Ice-slurry. On the horizontal axis, C denotes cycling and R denotes running within the blocks. Mean values and SD are shown.
(EPS)

S4 Fig Heart rate (beats.min^-1^) during warm-up, exercise and recovery for trials with the ingestion of Neutral water, Cold water and Ice-slurry. On the horizontal axis, C denotes cycling and R denotes running within the blocks. Mean values and SD are shown.
(EPS)

S5 Fig (A) Rating of thermal sensation, (B) rating of thermal comfort and (C) rate of perceived exertion during trials with the ingestion of Neutral water, Cold water and Ice-slurry. ^a^ Significantly different from Neutral water (P<0.05). Mean values and SD are shown.
(EPS)

S1 Table Core temperature and Heart rate for cycling, running and blocks when absorbing neutral water/menthol, cold water/menthol and ice-slurry/menthol. Mean ± SD are shown.
(EPS)
